# Antibiotic treatment failure of uncomplicated urinary tract infections in primary care

**DOI:** 10.1186/s13756-023-01282-4

**Published:** 2023-08-01

**Authors:** Sky Wei Chee Koh, Tracy Si Min Ng, Victor Weng Keong Loh, Jun Cong Goh, Si Hui Low, Wei Zhi Tan, Hung Chew Wong, Pradeep Durai, Louisa Jin Sun, Doris Young, Paul Anantharajah Tambyah

**Affiliations:** 1 National University Polyclinics, National University Health System, Singapore, Singapore; 2 Division of Family Medicine, Yong Loo Lin School of Medicine, National University of Singapore, Singapore, Singapore; 3 Department of Family Medicine, National University Health System, Singapore, Singapore; 4 Emergency Department, Southern Adelaide Local Health Network, Adelaide, Australia; 5 School of Biological Sciences, Nanyang Technological University, Singapore, Singapore; 6 Biostatistics Unit, Yong Loo Lin School of Medicine, National University of Singapore, Singapore, Singapore; 7 Division of Urology, Department of General Surgery, Ng Teng Fong General Hospital, Singapore, Singapore; 8 Infectious Diseases, Alexandra Hospital, National University Health System, Singapore, Singapore; 9 Department of Medicine, Yong Loo Lin School of Medicine, National University of Singapore, Singapore, Singapore; 10 Division of Infectious Disease, University Medicine Cluster, National University Hospital, Singapore, Singapore

**Keywords:** General practice, Primary care, Urinary tract infections, Antibiotic, Treatment failure, Effectiveness, Antimicrobial resistance, Amoxicillin-clavulanate, Singapore

## Abstract

**Background:**

Higher resistance rates of > 20% have been noted in *Enterobacteriaceae* urinary isolates towards ciprofloxacin and co-trimoxazole (C + C) in Singapore, compared with amoxicillin-clavulanate and nitrofurantoin (AC + N). This study examined if treatment failure varied between different antibiotics, given different resistant rates, for uncomplicated urinary tract infections (UTIs) managed in primary care. We also aimed to identify gaps for improvement in diagnosis, investigations, and management.

**Methods:**

A retrospective cohort study was conducted from 2019 to 2021 on female patients aged 18–50 with uncomplicated UTIs at 6 primary care clinics in Singapore. ORENUC classification was used to exclude complicated UTIs. Patients with uncomplicated UTIs empirically treated with amoxicillin-clavulanate, nitrofurantoin, ciprofloxacin or co-trimoxazole were followed-up for 28 days. Treatment failure was defined as re-attendance for symptoms and antibiotic re-prescription, or hospitalisation for UTI complications. After 2:1 propensity score matching in each group, modified Poisson regression and Cox proportional hazard regression accounting for matched data were used to determine risk and time to treatment failure.

**Results:**

3194 of 4253 (75.1%) UTIs seen were uncomplicated, of which only 26% were diagnosed clinically. Urine cultures were conducted for 1094 (34.3%) uncomplicated UTIs, of which only 410 (37.5%) had bacterial growth. The most common organism found to cause uncomplicated UTIs was *Escherichia coli* (64.6%), with 92.6% and 99.4% of isolates sensitive to amoxicillin-clavulanate and nitrofurantoin respectively. Treatment failure occurred in 146 patients (4.57%). Among 1894 patients treated with AC + N matched to 947 patients treated with C + C, patients treated with C + C were 50% more likely to fail treatment (RR 1.49, 95% CI 1.10–2.01), with significantly higher risk of experiencing shorter time to failure (HR 1.61, 95% CI 1.12–2.33), compared to patients treated with AC + N.

**Conclusion:**

Treatment failure rate was lower for antibiotics with lower reported resistance rates (AC + N). We recommend treating uncomplicated UTIs in Singapore with amoxicillin-clavulanate or nitrofurantoin, based on current local antibiograms. Diagnosis, investigations and management of UTIs remained sub-optimal. Future studies should be based on updating antibiograms, highlighting its importance in guideline development.

## Background

Urinary tract infection (UTI) is a common presentation in primary care, with half of all adult women experiencing 1 episode in their lifetime [1]. The most likely diagnosis is an uncomplicated urinary cystitis (caused by *Escherichia coli*), where a non-pregnant pre-menopausal female presents with typical urinary symptoms without risk factors highlighted in the ORENUC classification [2]. While 95% of general practitioners initiate empirical antibiotic therapy [3], urine cultures are not routinely performed as this is discouraged by guidelines worldwide [4, 5]. The choice of empirical antibiotic therapy depends on the most likely organism causing the infection and resistance patterns based on local antibiograms [6].

Although antibiotic treatment failure in uncomplicated UTIs may lead to complications such as pyelonephritis and sepsis, the impact of prescribing empirical antibiotics in relation to local antibiotic susceptibility patterns have not been well studied. While primary care studies worldwide detected a 10–18% antibiotic treatment failure rate for uncomplicated UTIs [7–10], differing outcome definitions made comparisons challenging [11]. Locally, the percentage of patients returning for treatment failure after initial antibiotic treatment of UTIs remained unknown. In-vitro microbiological resistance does not reliably predict clinical outcome and treatment failure rate [12, 13], hence echoing the need of such a study.

Primary care forms the foundation of the Singapore healthcare system, provided by 2000 private general practitioner clinics and 23 government subsidised primary care clinics (polyclinics) from 3 healthcare clusters. These public primary care clinics account for 21% of all primary care outpatient visits in the country. In Singapore, antibiotics are prescription-only drugs, and it will be illegal to acquire antibiotics from pharmacies without a legal prescription.

UTIs are among the top 10 principal causes of death in Singapore [14], and the 2nd most common indication for antibiotic therapy in primary care in 2021 [15]. A local study which showed high susceptibility of 283 urinary Enterobacteriaceae isolates to amoxicillin-clavulanate (86%) and nitrofurantoin (87%), with > 20% isolates resistant to ciprofloxacin and co-trimoxazole [16]. Despite this, the lack of antibiotic stewardship programmes in primary care [17] coupled with public antibiotic misinformation [18] and outdated guidelines [19] have resulted in rampant outpatient over-prescription of antibiotics in Singapore [20].

Therefore, this study aimed to investigate the likelihood of treatment failure for uncomplicated UTIs in primary care varied between different antibiotics, due to differences in antibiotic resistance patterns. Based on previous urinary antibiograms [16], we hypothesized that prescribing empirical antibiotics with higher resistance rates (ciprofloxacin and co-trimoxazole) (C + C) will lead to incomplete bacterial eradication, resulting in an increased risk of treatment failure, compared to antibiotics with lower resistance rates (amoxicillin-clavulanate and nitrofurantoin) (AC + N). This would be the first local study to compare choice of empirical antibiotic prescription on clinical outcomes and treatment failure rate. Secondary outcome of this study was to uncover areas of improvement in diagnosis, investigations, and management of uncomplicated UTIs in primary care, and to compare resistance patterns with previous local antibiogram studies. This would be of essence to assist in the development of UTI antibiotic guidelines, and solidify the need for routine community antibiograms in antimicrobial stewardship.

## Methods

### Study population and sampling

This was a 3-year retrospective cohort study involving 6 out of 7 public primary care clinics (polyclinics) within National University Polyclinics (NUP), using linked health record databases from 2 acute hospitals, National University Hospital (NUH) and Ng Teng Fong General Hospital (NTFGH) in Singapore, from 2019 to 2021. These clinics and hospitals are part of the National University Health Systems (NUHS) cluster, which serves 1.12 million residents in western Singapore. 1 clinic was excluded as it was opened in 2021. 1 clinic was previously using a different computer system and switched over to the same system during the study duration; therefore, some records could not be assessed.

### Study definition and parameters

The inclusion criteria were cases of uncomplicated UTIs, i.e., UTI in a healthy, non-pregnant, pre-menopausal female with anatomically and functionally normal urinary tract. Eligible patients were first identified by filtering all patient encounters with visit diagnoses labelled as disorder of kidney or ureter or UTI (ICD-10 codes N28.9, N39.0) that fell within the study period. Next, female patients aged 18–50 who were empirically treated with amoxicillin-clavulanate, nitrofurantoin, ciprofloxacin or co-trimoxazole during the same visit were selected. Incident UTI visit, defined as the visit occurring in a patient without a UTI-related diagnosis, or antibiotics prescribed for UTI in the preceding 90 days (to exclude patients on long term prophylaxis for recurrent UTI), were identified and included. This ensured that follow-up encounters that used similar visit diagnoses would not be double counted, and allowed tracking of patients whose antibiotic therapy was changed or extended in subsequent encounters from this incident UTI visit.

Exclusion criteria were patients on long-term antibiotic prophylaxis (≥ 4 weeks of a continuous antibiotic), complicated UTI (defined in European Association of Urology’s ORENUC classification system) [2], recurrent UTI (defined as ≥ 3 UTI visits which were treated with antibiotics within 1 year from incident UTI date), incomplete management, follow-up care or missing data. Episodes with a hospital discharge in the preceding 14 days were omitted to exclude hospital acquired infections. Individual case notes review was then performed to ensure that all cases fell within the inclusion and exclusion criteria, and were eligible for follow-up.

For eligible cases, variables such as patient age, race, presence of chronic diseases (pre-diabetes, diabetes, chronic kidney disease), urinary investigations, culture and susceptibility test results, antibiotic treatment and visit dates were extracted. Most of the urine cultures were performed and reported according to standards set by Clinical and Laboratory Standards Institute (CLSI); 1 clinic which was previously under a different public healthcare institution used European Committee on Antimicrobial Susceptibility Testing (EUCAST) standards. Case notes review was performed manually; cases with inappropriate diagnoses coding, erroneous antibiotic prescriptions, follow-up consultations were excluded. Discrepancies, issues with inclusion and exclusion criteria or determination of treatment failure were discussed at study team panel discussions, de-conflicted by 2 physicians until consensus was reached. Patients who were treated with amoxicillin-clavulanate and nitrofurantoin (AC + N), and co-trimoxazole and ciprofloxacin (C + C) were split into 2 groups. This was done to increase the statistical power, and because of potential considerations of recommending AC + N in future antibiotic guidelines for UTI.

These patients were followed up for 28 days from day of incident visit, via case notes review. Follow-up period begun from the earliest study start date and ended on the study end date, the day patient deceased or 28 days after the incident UTI visit.

Treatment failure was defined as re-attendance to primary care or emergency department for urinary symptoms with a same-day antibiotic re-prescription, or hospitalization for complications of UTI (sepsis, pyelonephritis, acute kidney injury) within 28 days from incident UTI visit. We excluded patients who did not complete antibiotic due to side effects or were non-compliant. Primary outcome would be risk of, and time to, treatment failure, with an aim to quantify these differences between antibiotics with lower versus higher resistance rates. According to the Infectious Diseases Society of America and the European Society for Microbiology and Infectious Diseases guidelines, UTI relapse was defined as persistence of organism after 14 days despite antibiotic treatment [21]. We chose a 28-day follow-up for the definition of treatment failure to reduce the likelihood of missing late presentations beyond the 14-day mark that might still present with hospitalization because of complications from the initial UTI visit. This cut-off was also chosen by similar studies conducted worldwide, for easier comparison of our results with these studies.

To minimize risks of breach of confidentiality due to medical records access, all data was de-identified by a centralized trusted third party before analysis.

### Statistical analysis

Statistical analyses were performed with IBM® SPSS® Statistics Version 28.0 and SAS Version 9.4, with *p* value of < 0.05 in the two-sided test was considered as statistical significance. Descriptive statistics were performed; numerical variables were represented as mean with standard deviation (SD), median with minimum and maximum values, categorical variables as n (%). To emulate randomization, we used propensity score (PS) matching by including variables such as age, whether patient had presence of chronic diseases such as diabetes, pre-diabetes or chronic kidney disease, and availability and results of urine cultures. The urine cultures could grow bacteria which was susceptible to the antibiotic prescribed, indeterminate or resistant, or no cultures performed. We used a matching of 2:1 ratio for AC + N:C + C to improve statistical efficiency as the number of patients treated with AC + N was much larger compared to those treated with C + C. This allowed us to retain as many patients on AC + N as possible (84.3%). Modified Poisson regression was used to determine the association between antibiotic prescribed and treatment failure. Cox proportional hazard regression accounting for matched data was used to examine whether there is any significant difference in time to treatment failure between patients on AC + N and patients on C + C. Patients who did not receive a second antibiotic were censored on the 28th day. To maintain statistical independence, only the incident visit was included in the analysis.

## Results

**Fig. 1:**
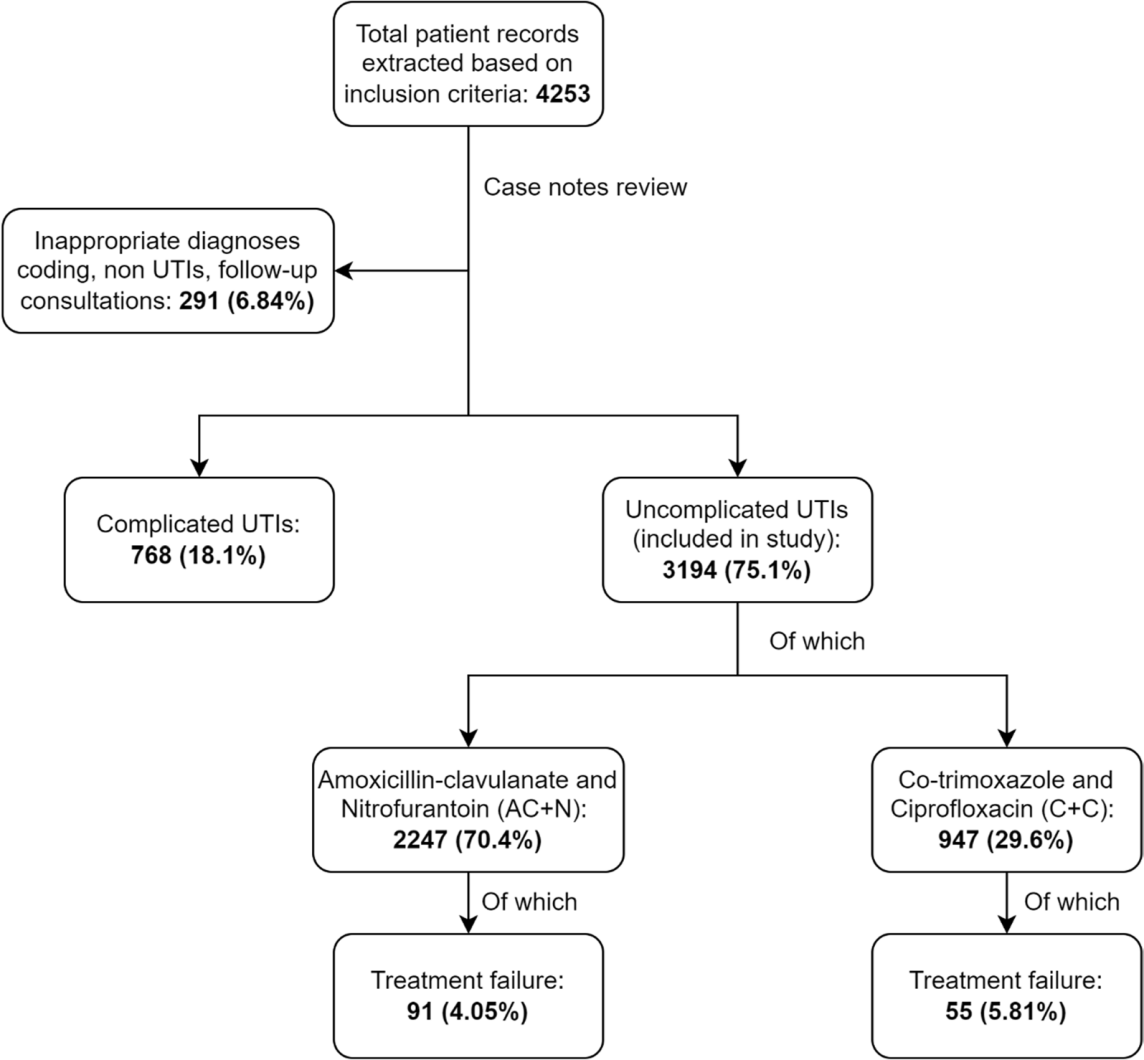
Study characteristics of UTI patients and treatment failure in both antibiotic groups, 2019–2021

4253 patient records were extracted based on the inclusion criteria. After case notes review, 3194 patients were included in the study (Fig. 1).

From Table 1, the number of uncomplicated UTIs remained stable from 2019 to 2021. Urine microscopy (70.0%) was most used to assist in diagnosis of uncomplicated UTIs, while 26.0% were diagnosed clinically. Urine cultures were conducted for 1094 (34.3%) uncomplicated UTIs, of which only 410 (37.5%) had bacterial growth. The most common organism found to cause uncomplicated UTIs was *Escherichia coli* (64.6%). There was 92.6% amoxicillin-clavulanate and 99.4% nitrofurantoin susceptibility towards *Escherichia coli* respectively. This was compared to 80.0% and 82.5% for ciprofloxacin and co-trimoxazole. The most common empirical antibiotic initiated was amoxicillin-clavulanate (36.4%). Treatment failure occurred in 4.57% (n = 146) of patients.

**Table 1:** Characteristics of management of uncomplicated UTI patients

Study characteristics	Total
	(n = 3194)
*Primary care clinic, n (%)*	
Clinic A	585 (18.3)
Clinic B	592 (18.5)
Clinic C	542 (17.0)
Clinic D	647 (20.3)
Clinic E	730 (22.9)
Clinic F*	98 (3.1)
*Year, n (%)*	
2019	1009 (31.6)
2020	1035 (32.4)
2021	1150 (36.0)
*Investigations performed, n (%)*	
Urine microscopy (UFEME)	2236 (70.0)
Urine dipstick	74 (2.3)
Both	56 (1.8)
None	830 (26.0)
No urine cultures	2100 (65.7)
Negative urine cultures	684 (21.4)
Positive urine cultures	410 (12.8)
*Organisms in positive urine cultures, n (%)*	
*Escherichia coli*	343 (64.6)
*Streptococcus agalactiae*	63 (11.9)
*Klebsiella pneumoniae*	45 (8.5)
*Staphylococcus saprophyticus*	31 (5.8)
*Proteus mirabilis*	16 (3.0)
*Citrobacter koseri*	14 (2.6)
Others	32 (6.0)
*Escherichia coli antibiotic susceptibility*^*+*^*, n (%)*	
Ampicillin	195 (60.0)
Amoxicillin-clavulanate	300 (92.6)
Ciprofloxacin	260 (80.0)
Ceftriaxone	310 (95.4)
Co-trimoxazole	269 (82.5)
Gentamicin	306 (94.2)
Nitrofurantoin	318 (99.4)
Pip/Tazobactam	336 (98.2)
*Empirical antibiotics initiated, n (%)*	
Amoxicillin-clavulanate	1164 (36.4)
Nitrofurantoin	1083 (33.9)
Cotrimoxazole	534 (16.7)
Ciprofloxacin	413 (12.9)
*Treatment failure, n (%)*	
Primary care	123 (3.9)
Hospital	29 (0.9)

*Data from Clinic F from end 2020 onwards after transitioning onto new computer system

^+^Differences in antibiotic susceptibility percentages was due to differing laboratory susceptibility testing procedures in the 6 clinics

The age, race, presence of chronic diseases and urine culture results of studied patients before and after PS matching were described in Table 2. The mean age for uncomplicated UTIs was 33.06. Majority of patients were of Chinese race (58.0%), with 5.7% having chronic diseases.

**Table 2:** Baseline characteristics of uncomplicated UTI patients treated with AC + N and C + C before and after 2:1 PS matching

Baseline characteristics	Before PS matching			After PS matching	
	Total	AC + N	C + C	AC + N	C + C
	(n = 3194)	(n = 2247)	(n = 947)	(n = 1894)	(n = 947)
Mean age, y (SD)	33.06 (10.01)	32.98 (10.01)	33.24 (10.04)	33.21 (10.03)	33.24 (10.04)
Median age, y (min–max)	32 (18–50)	32 (18–50)	32 (18–50)	32 (18–50)	32 (18–50)
*Race, n (%)*					
Chinese	1852 (58.0)	1302 (57.9)	550 (58.1)	1108 (58.5)	550 (58.1)
Indian	391 (12.2)	269 (12.0)	122 (12.9)	226 (11.9)	122 (12.9)
Malay	653 (20.4)	462 (20.6)	191 (20.2)	386 (20.4)	191 (20.2)
Others	298 (9.3)	214 (9.5)	84 (8.9)	174 (9.1)	84 (8.9)
Presence of diabetes, pre-diabetes or CKD, n (%)	183 (5.7)	133 (5.9)	50 (5.3)	99 (4.8)	50 (5.3)
*Urine culture results, n (%)*					
Bacteria susceptible to antibiotic prescribed	354 (11.1)	276 (12.3)	78 (8.2)	156 (8.2)	78 (8.2)
Bacteria indeterminate or resistant to antibiotic prescribed	56 (1.8)	37 (1.6)	19 (2.0)	36 (1.9)	19 (2.0)
No bacteria cultured	2784 (87.2)	1934 (86.1)	850 (89.8)	1702 (89.9)	850 (89.8)

After 2:1 PS matching, patients who were treated with C + C were 50% more likely to fail treatment compared to patients who received AC + N, with RR = 1.49 (95% CI 1.10–2.01), *p* = 0.009.

**Fig. 2:**
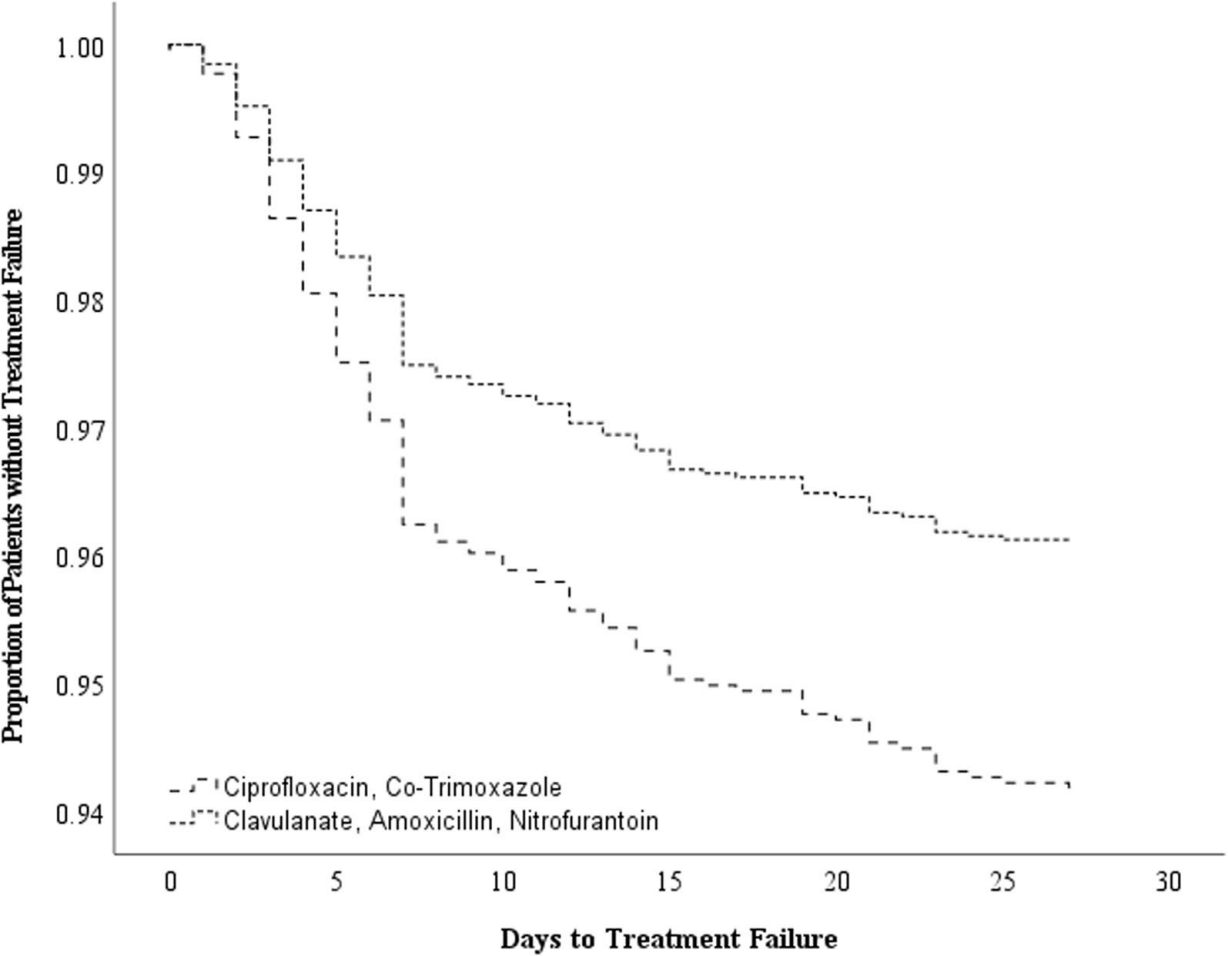
Cox regression for time to treatment failure between antibiotic groups after 2:1 PS matching

Patients who were treated with C + C had significantly higher risk of experiencing shorter time to failure compared to patients who were treated with AC + N (HR 1.61, 95% CI 1.12–2.33, *p* = 0.010) (Fig. 2).The mean days to treatment failure were 27.3 days (95% CI 27.1–27.5) for AC + N and 26.8 days (95% CI 26.5–27.1) for C + C respectively.

## Discussion

This retrospective cohort study was the first study conducted in Singapore specifically on uncomplicated UTIs in primary care. It not only showcased UTI management practices and antibiotic prescribing patterns, but also the importance of point-of-care urinary tests in UTI diagnosis, and updated local antibiograms for patients with uncomplicated UTIs. This would be essential in assisting the nation in guideline development.

Despite using a retrospective design, thorough case note review ensured stringent case selection and improved the study validity. While the classification of uncomplicated and complicated UTIs and incident UTI visits were often not clearly documented in case notes, a harmonized computer system allowed us to better ascertain UTI classification, incident visits and determine treatment failure cases. Nevertheless, we would assume that a small number of cases were lost to follow-up as they continued care with private general practitioner clinics or hospitals, which this study would not be able to assess. These could explain why the study treatment failure rate was about 5% for uncomplicated UTIs, which was significantly lower than similar studies conducted abroad (10–18%). Differences in outcome measures and criteria for treatment failure could have led to differences in treatment failure rates.

Our study found that 74% of uncomplicated UTIs were diagnosed with the help of point-of-care urinary investigations (urine microscopy or dipstick), and almost 35% had urine cultures performed (Table 1). This was contrary to practices recommended by international guidelines (NICE and EAU), where uncomplicated UTIs could be clinically diagnosed and empirically treated, with recommendations against the collection of urine cultures [4, 5]. We suspected that this variance in urinary investigations was stemmed from previously outdated local antimicrobial guidelines [19] and lack of widespread adoption of a more recently published recommendation [22]. This echoed the need for timely guideline review to ensure appropriate use of urinary investigations in the diagnosis of UTIs within primary care.

Our study also discovered similar bacteria profiles (64.6% grew *E*.* Coli*) and antibiotic susceptibilities (*E*.* Coli* was more resistant towards C + C than AC + N) compared to an earlier local study conducted in 2015–2016 [16]. Given that both studies were performed in the community setting, we speculated that the community resistance patterns for UTIs did not change drastically in the past few years. This study also supported the hypothesis of ‘culture-negative’ UTI previously suggested in the earlier local study [16], highlighting potential work in this area to determine the pathophysiology behind this phenomenon.

The reduction in ciprofloxacin prescriptions was also consistent with similar findings from another recent local study [15]. Moreover, patients treated with AC + N had about 1/3 less risk of treatment failure compared to C + C, signifying the importance of routine updated community antibiograms with appropriate empirical antibiotic recommendations as the basis for antibiotic guidelines.

One of the key study limitations was treatment assignment bias due to confounding variables. While this could be mitigated using PS matching like what the authors have done, we realized that we would still not be able to account for significant unmeasurable and unobserved factors such as frequency of sexual activity, issues with urinary tract anatomy, hygiene practices, inherent physician and patient preferences for type of antibiotic (such as previous experience, patient expectations, and external influence) [15, 23]. Moreover, matching all these factors will require large numbers of samples with substantial overlap between groups; this would also under-represent and exclude some cases, making a study like this less realistic. While we could mitigate this by increasing study duration like other larger studies conducted abroad [24], changes in bacterial resistance patterns would make a longer longitudinal study impractical. Perhaps, a mixed methods prospective study will allow for collection of these variables to improve its validity.

While this study may be representative of the public primary care UTI landscape in Singapore, it cannot be generalized to entire primary care due to substantial (80%) numbers of private GP practices. Given its small sample size with low treatment failure rates, the results indicated that if AC + N rather than C + C were used as first-line for UTI treatment, then 57 patients would need to be treated to prevent 1 additional treatment failure. The clinical significance of this may be marginal. It should be remembered that 65% of UTIs in this study had no urine cultures, and the difference in failure rates in patients with culture-proven bacterial infection may be higher. Increasing sample size can improve precision and power of the study.

Some of the dispensed antibiotics may not have been taken by patients; as they had not initiated treatment, they were not counted as treatment failures in this study. Some patients with UTIs were treated using other antibiotics (such as cephalexin, fosfomycin) or came after initial private general practice visit, which were also excluded. Some also had inaccurate diagnoses coding and were not true UTIs. They constituted a small percentage (6.84%) and were excluded from this study. This study was retrospective, and patients were not randomized between treatments. Duration of treatment, which was not accounted for in this study, could also contribute to bacterial eradication and affect time to treatment failure. Antibiotic prescribed could also reflect symptom severity rather than physician preferences. Perhaps a randomized controlled trial could be considered in the primary care setting to fully address the abovementioned gaps and truly determine the impact of empirical antibiotics on treatment failure.

## Conclusion

In conclusion, this study ascertained the treatment failure rate for uncomplicated UTIs in Singapore, with reduced risk of and time to treatment failure for patients treated with AC + N when compared with treatment with C + C. Given the current local antibiograms, we recommend using amoxicillin-clavulanate or nitrofurantoin in the management of uncomplicated UTIs in Singapore. Areas of improvement included better recommendations on using urinary investigations in UTI diagnosis, need for updated urine culture and susceptibility profiles in guiding empirical antibiotic recommendations and increasing sampled population. This study had also showed us the difficulties in conducting a perfect study on uncomplicated UTIs in primary care, food for thought for the subsequent studies to come.

## Data Availability

The datasets used and/or analysed during the current study are available from the corresponding author on reasonable request.

## References

[1] Foxman B (2002). Epidemiology of urinary tract infections: incidence, morbidity, and economic costs. Am J Med.

[2] Johansen TEB, Botto H, Cek M (2011). Critical review of current definitions of urinary tract infections and proposal of an EAU/ESIU classification system. Int J Antimicrob Agents.

[3] Brooks D (1990). The management of suspected urinary tract infection in general practice. Br J Gen Pract.

[4] National Institute for Health and Care Excellence (NICE). Urinary tract infections in adults (QS90); NICE, United Kingdom. (2015). https://www.nice.org.uk/guidance/qs90. Accessed 24 Apr 2023.

[5] European Association of Urology. EAU Guidelines on Urological Infections; European Association of Urology. (2022). https://uroweb.org/guidelines/urological-infections. Accessed 24 Apr 2023.

[6] Thompson RL, Wright AJ (1998). General principles of antimicrobial therapy. Mayo Clin Proc.

[7] Ten Doesschate T, Groenwold RHH, Bonten MJM, van Werkhoven CH (2019). Effectiveness of extended- versus normal-release nitrofurantoin for cystitis: an instrumental variable analysis. J Antimicrob Chemother.

[8] Lawrenson RA, Logie JW (2001). Antibiotic failure in the treatment of urinary tract infections in young women. J Antimicrob Chemother.

[9] Goettsch WG, Janknegt R, Herings RMC (2004). Increased treatment failure after 3-days’ courses of nitrofurantoin and trimethoprim for urinary tract infections in women: a population-based retrospective cohort study using the PHARMO database. Br J Clin Pharmacol.

[10] Lin YS, Jan IS, Cheng SH (2017). Comparative analysis of the cost and effectiveness of generic and brand-name antibiotics: the case of uncomplicated urinary tract infection. Pharmacoepidemiol Drug Saf.

[11] Neill R, Gillespie D, Ahmed H (2022). Variation in antibiotic treatment failure outcome definitions in randomised trials and observational studies of antibiotic prescribing strategies: a systematic review and narrative synthesis. Antibiotics.

[12] Ferry S, Burman LG, Holm SE (1988). Clinical and bacteriological effects of therapy of urinary tract infection in primary health care: relation to in vitro sensitivity testing. Scand J Infect Dis.

[13] Davey P, Steinke D, MacDonald T, Phillips G, Sullivan F (2000). Not so simple cystitis: How should prescribers be supported to make informed decisions about the increasing prevalence of infections caused by drug-resistant bacteria?. Br J Gen Pract.

[14] Ministry of Health Singapore. Principal Causes of Death. Available online: https://www.moh.gov.sg/resources-statistics/singapore-health-facts/principal-causes-of-death. Accessed 10 Apr 2023.

[15] Koh SWC, Lee VME, Low SH (2023). Prescribing antibiotics in public primary care clinics in singapore: a retrospective cohort study. Antibiotics.

[16] Ho HJ, Tan MX, Chen MI, Tan TY, Koo SH, Koong AYL, Ng LP, Hu PL, Tan KT, Moey PKS, Koh EYL, Wong CS, Lye DC, Tan NC (2019). Interaction between antibiotic resistance, resistance genes, and treatment response for urinary tract infections in primary care. J Clin Microbiol.

[17] Chua AQ, Kwa AL, Tan TY, Legido-Quigley H, Hsu LY (2019). Ten-year narrative review on antimicrobial resistance in Singapore. Singap Med J.

[18] Pan DST, Huang JH, Lee MHM, Yu Y, Chen MI-C, Goh EH, Jiang L, Chong JWC, Leo YS, Lee TH, Wong CS, Loh VWK, Poh AZ, Tham TY, Wong WM, Lim FS (2016). Knowledge, attitudes and practices towards antibiotic use in upper respiratory tract infections among patients seeking primary health care in Singapore. BMC Fam Pract.

[19] Ministry of Health Singapore. Clinical Practice Guidelines: Use of Antibiotics in Adults. 2006. Available online: https://www.moh.gov.sg/docs/librariesprovider4/guidelines/cpg_use-of-antibiotics-in-adults-feb-2006f6e0e4f9dad34a1f8f07666a05ec59a1.pdf?sfvrsn=244726fe_0, Accessed 10 Apr 2023.

[20] Lee T-H, Wong JG, Lye DC, Chen MI, Loh VW, Leo Y-S (2017). Medical and psychosocial factors associated with antibiotic prescribing in primary care: survey questionnaire and factor analysis. Br J Gen Pract.

[21] Gupta K, Hooton TM, Naber KG, Wullt B, Colgan R, Miller LG (2011). International clinical practice guidelines for the treatment of acute uncomplicated cystitis and pyelonephritis in women: a 2010 update by the infectious diseases society of america and the european society for microbiology and infectious diseases. Clin Infect Dis.

[22] Wei Tan C, Chlebicki MP (2016). Urinary tract infections in adults. Singap Med J.

[23] Kotwani A, Wattal C, Katewa S, Joshi PC, Holloway K (2010). Factors influencing primary care physicians to prescribe antibiotics in Delhi India. Fam Pract.

[24] Daneman N, Chateau D, Dahl M (2020). Fluoroquinolone use for uncomplicated urinary tract infections in women: a retrospective cohort study. Clin Microbiol Infect.

